# Medical Opinion and Movement

**Published:** 1909-02-13

**Authors:** 


### MEDICAL OPINION AND MOVEMENT.

THE production of hæmostasis by elastic compression around the trunk of the body was first put forward by Dr. Momburg last year, and he published two cases in which the method had been successfully adopted in order to obtain hæmostasis in the lower part of the body. No accident or inconvenience was caused, although in one case the compression was kept up as long as 43 minutes. Since then several further cases have been reported. Dr. Momburg himself has reported one case in which both legs were crushed. The compression lasted 30 minutes. Dr. Rimann, of Leipzig, reports a second case of amputation at the hip joint, compression lasting 25 minutes. Dr. Axhousen reports a third case in which an extensive operation on the pelvic viscera for tubercular lesions was carried out under compression by this method, lasting 45 minutes. Finally, Dr. Willems has tried the method in four cases, and in one only he failed to obtain satisfactory hæmostasis owing to scoliosis. Otherwise in each case the hæmostasis is said to have been complete and without any accident of any kind. It is advisable to obtain anæsthesia first and then an elastic tube is wound round the waist two or three times till the femoral pulse can no longer be felt.

---

TWO dangers in this method naturally suggest themselves: an injurious effect of the compression upon the abdominal viscera, especially the intestines and ureters, and cardiac trouble likely to follow removal of the compression and the consequent sudden fall in blood pressure. As a safeguard against the latter it is proposed that before removal of the tube compressing the trunk elastic bandages should be applied to the lower limbs, and then should be removed successively after releasing the elastic tube. Dr. Momburg proposes the following method in cases of operation upon the pelvis: The lower limbs are first encompassed by an Esmarch's bandage from below upwards. Elastic compression is then applied to the trunk and the bandages are removed. The patient is placed in an inclined position, with the trunk raised and the limbs lowered. In this way all the blood remaining in the vessels of the pelvis passes into the lower limbs. Two elastic bandages are then applied at the upper end of the thighs to prevent a return flow when the patient is placed in a horizontal position, and the intermediate zone—that is, the whole pelvic region between the elastic compression of the trunk and that of the thighs—remains quite bloodless.

---

DIEULAFOY has some time back called attention to the fact that some cases regarded as appendicitis are really examples of membranous colitis, in which operation is generally of little benefit, and certainly not commensurate with the risk inseparable from operation. Professor Wilms, of Basel, now maintains that in certain cases of so-called chronic appendicitis the condition is not due to any affection of the appendix itself, but to the presence of a long and movable cæcum, which gives rise to pain owing to traction upon its mesentery and upon that of the appendix. The patients complain of pain or intermittent sensations of discomfort in the right side of the abdomen, which are rendered worse by constipation. The pain radiates, and there is generally some sensitiveness to pressure around McBurney's point. In diagnosing this condition Professor Wilms lays special stress upon the presence of a loud gurgling sound on palpating the right side of the abdomen, and also to the possibility of moving the elongated cæcum to and fro with the fingers. Occasionally, if the cæcum is contracted, a tumour-like resistance is encountered. The cæcum is to be regarded as loose and elongated when it can be drawn *en masse* up to the wound. To relieve the condition Professor Wilms fixes the cæcum in a new position by forming a sort of pocket for it in the pelvic peritoneum. He has operated in this way in forty cases with complete recovery, but he removed the appendix at the same time. In order, however, to eliminate the possibility that the cure was effected by this latter procedure alone he has removed the appendix only in a number of cases of the same kind, with the result that the symptoms have recurred.

---

THE new principle now adopted by several Continental surgeons of allowing patients to sit up out of bed and even walk about within the first week after laparotomy has already been commented upon in these columns. The method appears to have gained several adherents recently, and general satisfaction is expressed with the results. As might have been expected, however, certain complications are likely to arise, especially thrombosis, which figures rather highly in a series of 100 cases reported by Pfannenstiel. These 100 patients were allowed to get up between the first and seventh day, half of them on the third or fourth day. The author expresses himself as thoroughly satisfied with the rapidity of convalescence, and the patients themselves are said generally to have appreciated the method of treatment. Some of them declared spontaneously that pains felt while lying down disappeared when they were up. They could walk without the feelings of weakness and giddiness which usually follow a prolonged stay in bed. In these 100 cases there were, however, three cases of thrombosis. These certainly appear to have been to some extent attributable to the condition of the patient before operation. One case was that of a woman aged 66 with an ovarian cyst. There was œdema of the lower limbs before operation. She was allowed to get up early for fear of hypostatic pneumonia. The second was that of a patient who had been rendered extremely anæmic by a hæmorrhagic fibroid, and before operation had a temperature of 38.4° C.; and the third was also a fibroid complicated with suppurative salpingitis and fever. Whatever the advantages of the method may be it is certainly advisable to bear in mind this possibility of thrombosis and also that of embolism.

In *L'Echo Médical des Cevennes*, Dr. Reynaud discusses the treatment of chronic gonorrhœa. He is of opinion that it is dangerous in many cases to treat the disease by preparations of the nature of sandal-wood oil, copaiba, etc., for once the purulent discharge is suppressed, the tendency to stricture continues, despite this form of drug treatment. He is likewise unfavourably impressed with the effects of the ordinary treatment by injections of caustics, disinfectants, astringents, etc. These do not act on the pathological cause of the disease. He is, on the other hand, favourably impressed by the results of treatment as instituted by Motz. Glycerine suppositories are placed in the urethra and retained there as long as possible. The mucous membrane of the canal is thus made to desquamate, with the result that epithelium of pathological origin is cast off and replaced later by one of more normal type. The glands of Liltré are subsequently squeezed out by massage of the part, and the microbes destroyed by injections of weak solutions of oxycyanate of mercury or permanganate of potash. Massage is next applied in the region of Cowper's glands and the prostate, and the injections repeated. The author is of opinion that this method of treatment is extremely effective in avoiding the formation of inflammatory strictures. It cannot be said that the treatment is in any way unreasonable, for good effects have often been obtained from the passage of a silver sound of large calibre in cases of chronic gleet. The sound in such cases takes the place of massage, and by reason of its calibre and hardness presses out the contents of the glands and crypts of the spongy urethra, and with them many of the offending organisms.

---

In the *Bulletin Médical* Professor Chantemesse discusses the prophylaxis of phlebitis and embolism by the administration of citric acid. To measure the coagulability of the blood a few drops of it are added to an equal quantity of a solution of potassium oxalate of known strength. The degree of concentration necessary to prevent the formation of a clot gives the exact measure of the coagulability of the blood. When this has been determined the patient is treated with citric acid until the blood has been brought back to a state of normal coagulability as determined by a series of examinations by the oxalate method. This method can also be used to ascertain the quantity of calcium chloride which it is necessary to administer in cases where the coagulability of the blood is below normal. The author recommends that both drugs should be given in large quantities of water, and that the daily dose of citric acid should be from 12 to 18 grams, that of calcium chloride being from 4 to 6 grams.

---

Von Sury, basing his conclusions on the results of 200 post-mortem examinations at the Institute of Vienna, as also on a study of the literature of the subject, denies that hypertrophy of the thymus is a cause of sudden death in children. The lumen of the trachea in the new-born has normally an oval shape on transverse section, and such flattening as is sometimes seen is really due to the impression of the vessels. Sudden death in cases where this flattening is found can always be explained by natural causes, such as broncho-pneumonia, enteritis, etc., which may have been overlooked at the autopsy. It is true that partial or complete extirpation of the thymus will relieve respiratory troubles, stridor, etc., in small children, but an explanation of this fact has not yet been found. The author points out that in none of the published cases of sudden death from hypertrophy of the thymus have the contents of the pulmonary alveoli been examined with a view to determining the possibility of asphyxia being due to aspiration of fluids, such as meconium, etc. He points out also that involution of the organ only commences about the time of puberty, and that it is not rare to discover a large thymus in the adult. He maintains that the expression "death from hypertrophy of the thymus" only hides the fact that there are not sufficient data for a more precise diagnosis.

---

The multiplicity of the methods of treatment of whooping-cough is a proof of the ineffectiveness of any particular one of them; and it is perhaps justifiable to suppose that this disease, like pneumonia, is merely a clinical conception corresponding to a variety of infectious states. If this view be correct it is easy to understand why pertussis varies so greatly in contagiousness and intensity, and why the results of drug treatment are so inconstant. Czerny, of Breslau, suggests that the disease is a neurosis superimposed on to a lesion of the respiratory mucous membrane, and is of opinion that the neurosis is really the more important part of the affection. He maintains that it is possible, after seeing the children of a given school, to predict which of them will be attacked the most seriously, for it is always those of neuropathic family antecedents who are so attacked. In the treatment of whooping-cough the author directs all his attention to the nervous system. He maintains that suggestion plays a large part in aggravating the cough in children suffering from mild attacks. It is, therefore, wrong to place such a child in the company of others who are coughing much. All that is necessary as regards isolation in such cases is to keep the patient in bed. There is then no risk of infecting other children. Every care should be taken to avoid agitating the child, and no discussion of the malady should be permitted in its presence.

---

The well-known good effect of a change of surroundings in whooping-cough is thus due on this hypothesis to the child's removal from a neuropathic circle which has been exercising a deplorable influence on its psychical condition. The powerful influence of suggestion explains also the failure of therapeutic measures in the case of young children who escape the influence of the spoken word. It is difficult to specify beforehand what type of drug will exercise curative suggestion. In some cases bitters, such as quinine, act extremely well. In other cases the desired effects can be produced by hydrotherapy, accompanied by nasal lavage and inhalations. In all cases where one type of treatment fails it should be immediately replaced by another of quite different type; and it is not necessary that the affected organs should be the ones treated. Good effects have been obtained by narcosis, vaccination, etc. The author insists that the practitioner must at all costs inspire the *entourage* of the patient with the greatest faith in the efficacy of the drug prescribed, even though repeated failure in his other cases have made him incline somewhat to a state of therapeutic nihilism.

---

Pulmonary embolism following operation is so sudden and tragic a complication that its occurrence is always provocative of reflection upon its cause. Dr. Gibson, of New York, has had five deaths in the last eight years from this trouble, and he sums up in the *Medical Record* his conclusions, and those expressed at the German Surgical Congress of 1908, upon the subject. Patients between forty and sixty are the most liable to this form of embolism—or, at least, are most likely to die of it. It is suggested that in the young the yielding elasticity of the vessel may allow the blood to push its way past an arrested clot. Practically all cases follow operations "below the belt," and this indicates that local conditions demand careful study in the search for the causation of such embolisms. Last of all, Dr. Gibson holds that there is no justification for rushing patients out of bed as a routine measure of prophylaxis, though he admits that in individual cases and conditions a pre-existent or suspected tendency to stagnation or coagulation should be counteracted by this and other measures. It is a little surprising thus to find that some American surgeons regard early rising after operations as a preventive of embolism; for over here it is commonly taught that injudicious movement is precisely that which is most likely to dislodge a recent clot. However, it is satisfactory that Dr. Gibson condemns this method of treatment, even if on grounds somewhat different to those which in Britain are considered to contra-indicate it.

---

The same condition is dealt with in the *Practitioner* by Mr. Leonard Bidwell, who says it is unpreventable, since the active cause is not known. He does not agree with the opinion held by some surgeons, both in Britain and America, that all cases of thrombosis are necessarily septic in origin; but is in favour of protecting the wound edges during laparotomy by gauze pads placed under the retractors, which has been advocated on the supposition that bruising of the deep epigastric veins is a frequent excitant of femoral thrombosis. At the same time this author points out that some cases of sudden death after operation or parturition may be due not to embolism, but to thrombosis of the pulmonary artery; and he gives details of eight such cases from his own practice. The anæsthetic used has apparently no influence on this form of thrombosis, although the onset of symptoms is as a rule within a very few days of the operation, thus differing from what is found in embolism, which is commonest from the tenth to fourteenth days. Patients suffering from fibroids are peculiarly liable both to thrombosis and embolism, possibly because of anæmia due to metrorrhagia, possibly because of an increase of calcium selts in the blood as shown by the tendency to calcareous degeneration. Finally, Mr. Bidwell remarks that the occurrence of pulmonary embolism may be less infrequent than is supposed, owing to non publication of statistics.

---

An interesting case of multiple suppurative arthritis following ophthalmia neonatorum is reported by Dr. Leslie Buchanan in the *Ophthalmoscope*. His patient was a baby, three weeks old when first seen, which exhibited the first symptoms of conjunctivitis as late as ten days after its birth. Both eyes became affected, though under treatment recovery was eventually complete in each. On the thirty-second day of the disease, and twenty-two days after treatment began, both eyes being then nearly well, it was noticed that the left forefinger and right great toe were much swollen. Subsequently an abscess also formed in the palm of the right hand; and all three lesions were treated by operation. A fortnight after, the right knee swelled but did not suppurate, and recovered without surgical interference. All the joints thus affected were restored to perfect function, and from those which were opened the gonococcus was recovered; as it was also from the pus of the original ophthalmia, together with an unidentified bacillus which is believed not to have been the Klebs-Lœffler bacillus, though a membranous formation not unlike that of diphtheritic conjunctivitis was noted. Dr. Buchanan emphasises the involvement of the small joints as a most unusual feature of gonococcal arthritis. The patient was at one time very seriously ill, and the prognosis was considered grave: but the result was satisfactory beyond the most sanguine expectation.

---

Professor William R. Smith, the Principal of the Royal Institute of Public Health, contributes a paper on "The Poisonous Gaseous Emanations of Ferro-Silicon," to the "Journal of the Royal Institute of Public Health." He states that the result of the inquiry into the cause of death of the five Russian immigrants on board the steamship *Ashton*, conducted for the most part at the laboratories of the Institute, shows that they died from the effects of gaseous emanations from the cargo (ferro-silicon) mainly attributable to the presence of phosphoretted hydrogen. So far as he can ascertain this is the first occasion in this country in which deaths have been known to be attributable to this cause. As ferro-silicon is a substance the manufacture of which on a large scale has only been undertaken within the last few years, references in scientific literature to the poisonous nature of its gaseous emanations are very scanty. In conclusion, Professor Smith announces that, since the pathological effects of such poisonous gases as phosporetted hydrogen and the like are at present so little understood, it has been determined to undertake further experiments in connection with the whole subject.

